# Contextual Variables Affect Running Performance in Professional Soccer Players: A Brief Report

**DOI:** 10.3389/fspor.2021.778813

**Published:** 2021-12-13

**Authors:** Diêgo Augusto, João Brito, Rodrigo Aquino, Pedro Figueiredo, Fabio Eiras, Márcio Tannure, Bruno Veiga, Fabrício Vasconcellos

**Affiliations:** ^1^Institute of Physical Education and Sports, State University of Rio de Janeiro, Rio de Janeiro, Brazil; ^2^Laboratory of Soccer Studies (LABESFUT), Rio de Janeiro, Brazil; ^3^Post-graduate Program in Exercise and Sport Sciences, Rio de Janeiro, Brazil; ^4^Portugal Football School, Portuguese Football Federation, Oeiras, Portugal; ^5^Department of Sports, Center of Physical Education and Sports (CEFD), Federal University of Espírito Santo, Vitória, Brazil; ^6^Research Center in Sports Sciences, Health Sciences and Human Development, CIDESD, Maia, Portugal; ^7^CIDEFES, Universidade Lusófona, Lisboa, Portugal; ^8^Independent Researcher, Rio de Janeiro, Brazil

**Keywords:** association football, time-motion analysis, game-analysis, team sports, tracking

## Abstract

This study aimed to investigate the effects of contextual variables on running performance in Brazilian professional soccer players. Twenty male players from one club participating in the 1st Division of the Brazilian soccer championship were analyzed during 35 matches. Global Positioning System was used to determine total distance (TD) covered, distance covered and actions in high intensity and sprinting, and the number of accelerations, and decelerations. The independent variables used were match location, match outcome, opposition ranking, change of head coach, and distance traveled to play the matches. Total distance was higher in a way than home matches (9,712 vs. 9,533 m; *p* ≤ 0.05), and losses than draws and wins (9,846 vs. 9,400 vs. 9,551 m; *p* ≤ 0.05), whereas distance in sprinting was higher in draws than losses (203 vs. 175 m; *p* ≤ 0.01). Changing the head coach during the season resulted in overall lower distance covered in high intensity, sprinting, high-intensity actions (*p* ≤ 0.01), and decelerations (*p* ≤ 0.05). Higher values for distance covered in sprinting and high intensity were found in matches without travel compared to those with long-travel (*p* ≤ 0.05). Overall, running performance was affected by the location, match outcome, change of head coach, and distance traveled during the season.

## Introduction

Match analysis has become a critical process in the prescription of specific and representative training in soccer. For this, running performance during the game (i.e., match running performance) has been monitored to access players' external load. Recently, the interest of researchers and practitioners in factors that influence running performance has grown (Castellano et al., 2011; Lago-Peñas, 2012; Moalla et al., 2018), with emphasis on contextual variables, including match location, match outcome, competitive level, and the quality of opponents (Paul et al., 2015; Liu et al., 2016; Aquino et al., 2020).

Regarding the match outcome, a greater number of high-intensity actions in matches won compared with the losses has been reported (Aquino et al., 2017a). Also, Folgado et al. (2014) showed that players covered shorter total distances (TDs) in matches played against low-level opponents compared with higher-level opponents. On the other hand, Rampinini et al. (2007) observed greater TD and distance covered at high intensity in matches against strong teams. This divergence reinforces the need for research addressing this topic in different leagues worldwide.

Similarly, some studies have reported that running performance is influenced by match location. Almeida et al. (2014) and Paraskevas et al. (2020) showed that players covered similar TDs comparing home and away matches. In contrast, Lago et al. (2010) showed higher values for TD covered in home matches. Most studies were developed involving European clubs (Lago et al., 2010; Almeida et al., 2014). Though, Dellal et al. (2011) noted that different leagues and cultures may also influence athletes' physical and technical performance. Therefore, research involving elite players worldwide may increase the plurality of the body of knowledge around this topic.

Also, coach replacement is a common phenomenon in soccer. As a recent example, in the 2019 elite Brazilian championship, 24 coach changes occurred throughout the 38 rounds. Coach replacement may result in changes in the game model, work methodology, and athletes' incentive level (Tozetto et al., 2019). These factors can directly link to changes in physical performance during matches. Guerrero-Calderón et al. (2021) showed that players modified behavior in relation to physical performance after changing coaches. Therefore, it might be necessary to understand the effects of coach replacement on match running performance in soccer.

Another common particularity of professional soccer is the need to frequently travel long distances to play matches and its potential impact on the physical demands (Watanabe et al., 2017). For instance, Brazil is considered a country of continental extension, which is approximately 8.5 million km^2^, and teams must travel weekly to play matches, which might interfere with players' recovery periods (Waterhouse et al., 2004; Fullagar et al., 2016). Therefore, it is common for a team from the southern region to travel more than 3 km to play a match. Although previous studies in Australian soccer showed that travel influenced physical performance and training (Fowler et al., 2014, 2015b), this contextual factor remains relatively unexplored in the literature.

Hence, the present brief report aimed to investigate the effects of match location, match outcome, quality of opposition, change of head coach, and distance traveled to play on running performance in Brazilian professional soccer players.

## Methods

The present investigation had a retrospective observational design. Overall, the 1^st^ Division of the Brazilian soccer championship consists of a double round-robin system, with 20 teams playing a total of 38 games (19 home and 19 away matches). This study analyzed 35 games from one club (*n* = 257 individual observations) during the 2017 season (May–December). Three matches were excluded from the analysis due to the large error measurements observed in data obtained by GPS. In total, 19 matches were played at home and 16 away, resulting in 13 wins, 11 draws, and 11 losses, with 49 goals scored and 38 goals conceded. The matches were played on natural grass pitches between 11:00 a.m. and 10:00 p.m.

### Participants

All outfield players were included in the study. Goalkeepers were excluded from the analysis. Only players who completed the entire match were considered for analysis (i.e., ≥ 90 min). In total, 22 players who are men (aged, 27 ± 5 years; height, 180.5 ± 6.9 cm; body mass, 74.8 ± 7.8 kg) were monitored. The study was conducted following the Declaration of Helsinki and was approved by the local university ethics committee (3.712.816).

### Dependent Variables

The players were monitored in all matches using GPS units (S5 Optimeye, Catapult Sports, Australia) with an acquisition frequency of 0 Hz and integrated with a 3D accelerometer of 100 Hz; excellent reliability has been reported (ICC = 0.77–1) (Nicolella et al., 2018). The players always used the same GPS units attached to a vest on the players' back. In addition, corporative software was used to calculate the TD covered, the number of actions and the distance covered at high-intensity (18.1–24 km/h) and sprinting (>24 km/h), and the number of accelerations (>2 m/s^2^) and decelerations (>2 m/s^2^).

### Independent Variables

Five contextual variables were considered: (i) match location (19 home matches and *n* = 144 individual observations; 16 away matches and *n* = 113 individual observations); (ii) match outcome (13 wins and *n* = 104 individual observations; 11 draws and *n* = 68 individual observations; 11 losses and *n* = 85 individual observations); (iii) quality of opposition, considering the final ranking of each team in the championship (1st to 6th place, *n* = 65 individual observations; 7th to 14th place, *n* = 113 individual observations; 15th to 20th place, *n* = 79 individual observations); (iv) change of head coach (17 matches for coach 1 and *n* = 126 individual observations;17 matches for coach 2 and *n* = 123 individual observations; 1 match was excluded from analysis as the team had a temporary head coach); (v) the distance traveled to play was calculated using a straight line between the team's local city and the match location city, using the GPS made available by GOOGLE™. The median distance traveled by the team throughout the competition was 520 km. This value was used to divide the trips into <520 km short trip and >520 km long trip. The matches were divided into 3 groups which were as follows: games without travel (games played in the same city regardless of the game's mandate, 21 matches, and *n* = 160 individual observations); games with short travel (games with travel <520 km, 7 matches, and *n* = 47 observations); games with long-travel (games with travel >520 km, 7 matches, and *n* = 50 observations).

### Statistical Analysis

Data are expressed as mean and (CI 95%). The non-normally distributed variables were corrected using a natural log transform. To account for the non-independence of data sampled from the same individuals across multiple matches separate linear mixed models were performed to compare (fixed effects) game location (home vs. away), change of head coach (coach 1 vs. coach 2), match outcome (win vs. draws vs. losses), and the opposition ranking (top six vs. middle eight vs. bottom six), traveled distance (without travel vs. short travel vs. long travel) with “athlete ID” included as a random effect. Furthermore, multiple comparisons were adjusted using the Tukey method. The *t*-statistics from the mixed models were converted to effect size correlations (Rosnow et al., 2000). The effect size (*r*) was classified as follows: trivial (*r* < 0.1), small (*r* = 0.1–0.3), moderate (*r* = 0.3–0.5), large (*r* = 0.5–0.7), very large (*r* = 0.7–0.9), and almost perfect (*r* > 0.9) (Hopkins et al., 2009). A significance level of *p* ≤ 0.05 was adopted. Data were analyzed with R software version 4.0.3.

## Results

**Figure 1:**
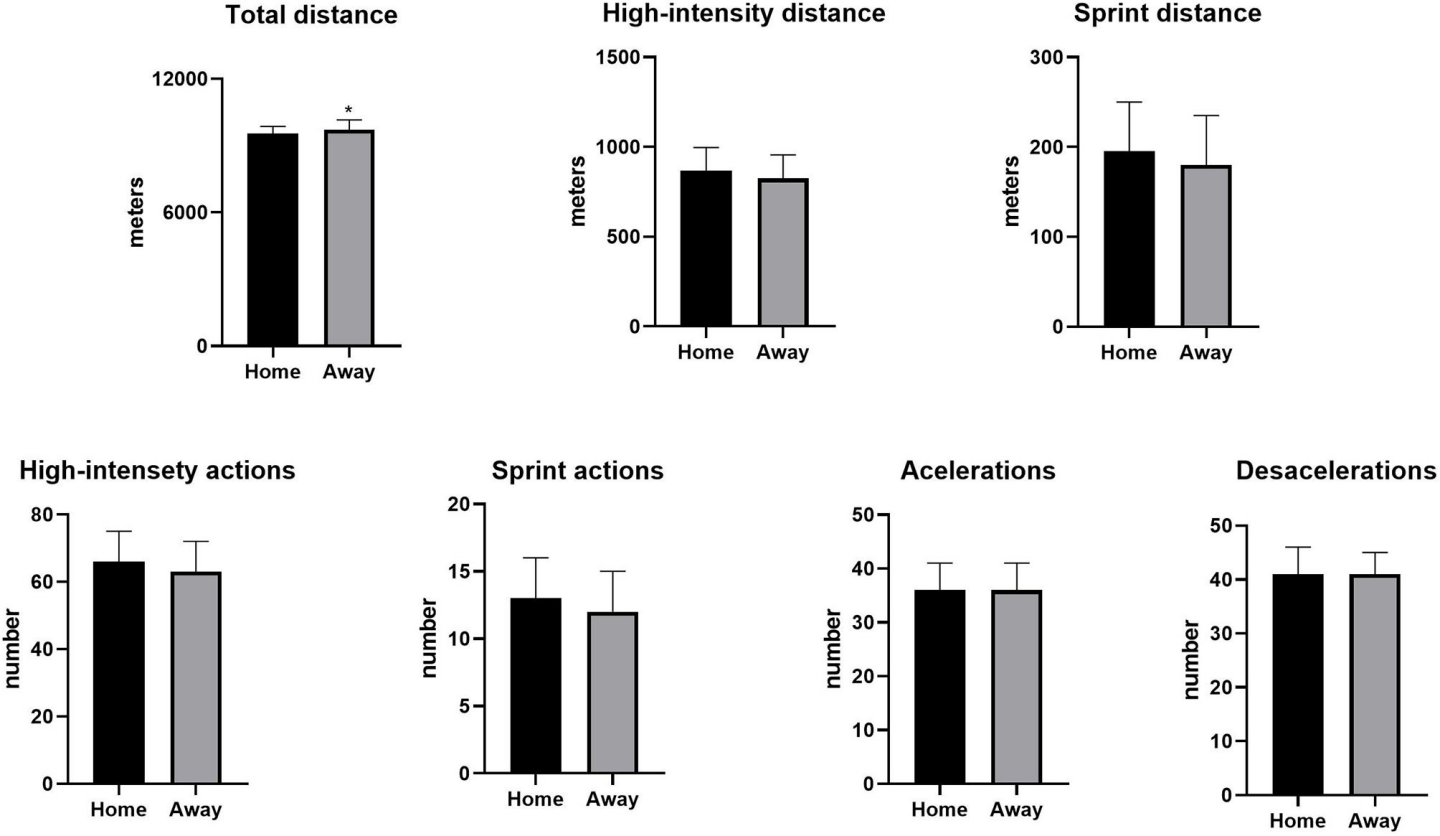
Running performance differences according to match location in Brazilian elite professional soccer players. *p ≤ 0.05.

Figure 1 shows the comparisons of running performance in relation to match location (home vs. away). Total distance covered was higher during away than home matches (*p* = 0.02; ES = 0.14). No significant differences were detected for any other performance variable.

**Figure 2:**
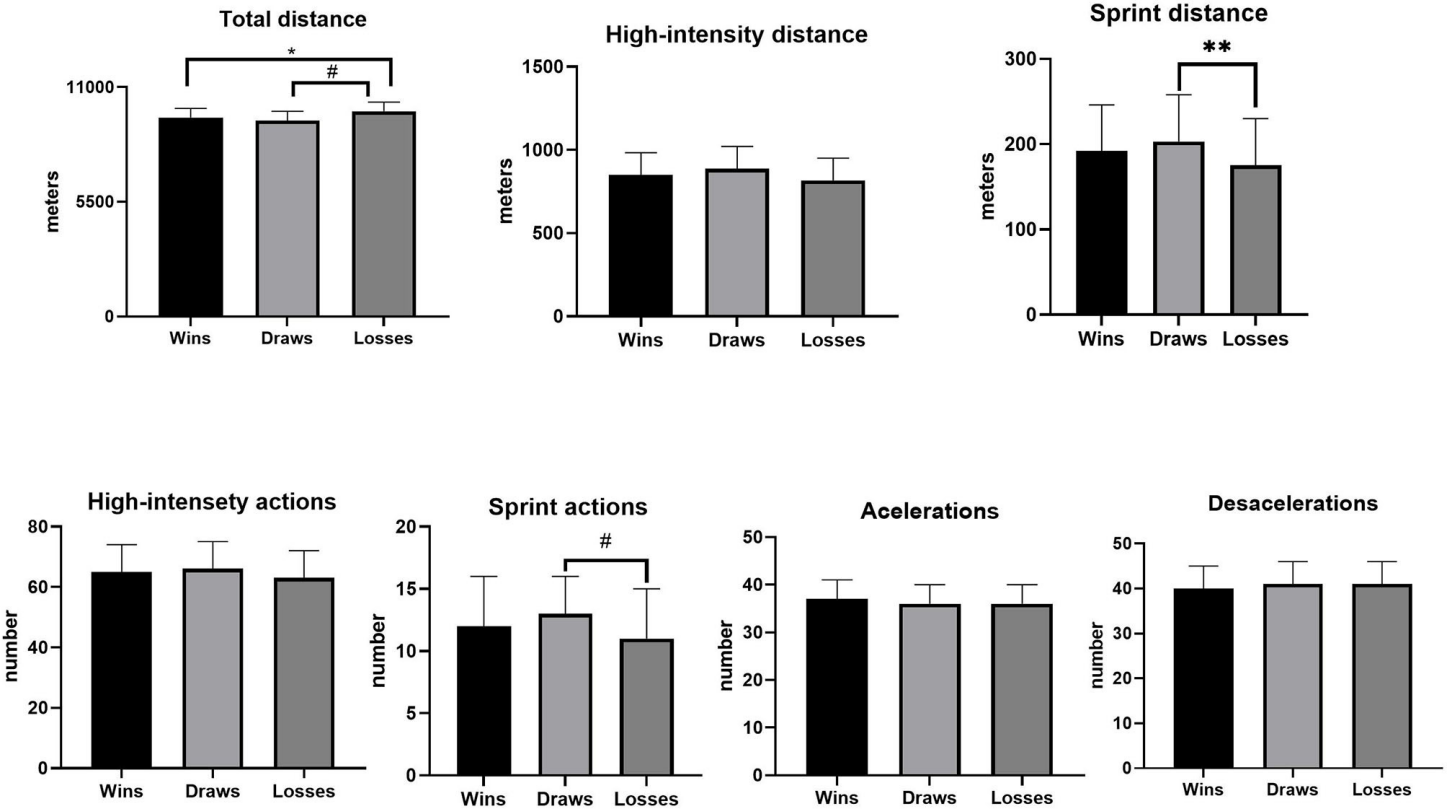
Running performance differences according to match outcome in Brazilian elite professional soccer players. *Loss > Draw; ^#^Loss > Win; **Draws > Loss.

Comparisons of running performance in relation to the match outcome showed that TD covered during matches lost was higher than for draws and wins (*p* = 0.03; ES = 0.27, and ES = 0.2, respectively; Figure 2). However, sprinting distance was higher in draws than matches lost (*p* = 0.03; ES = 0.45). No significant differences were detected for any other variable (*p* > 0.05).

**Figure 3:**
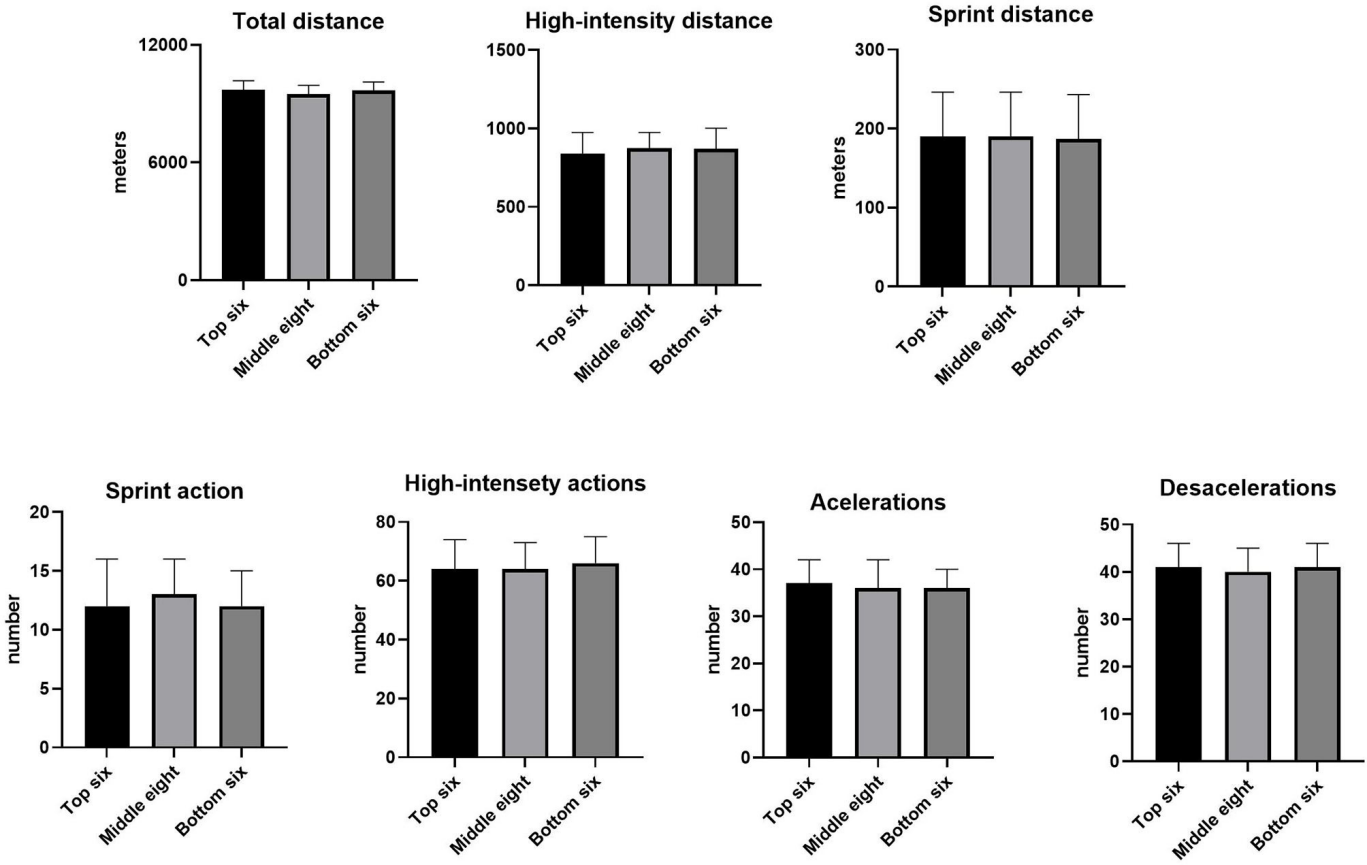
Running performance differences according to opposition ranking in Brazilian elite professional soccer players.

In addition, the comparisons of running performance regarding the opposition ranking are presented in Figure 3. No significant differences were detected for any performance variable between matches involving the top six, the middle eight, and the bottom six teams (*p* > 0.05).

Overall, running performance was affected by changing the head coach during the season. The distance covered in sprinting (202 m [CI 95% 147–258] vs. 178 m [CI 95% 123–234]) and high intensity (902 m [CI 95% 770–1,035] vs. 805 m [CI 95% 673–938]), the number of actions performed in high intensity (67 [CI 95% 53–76] vs. 63 [CI 95% 53–72]) and the number of decelerations (42 [CI 95% 37–47] vs. 40 [CI95% 35–45]) decreased when changing the coach (*p* < 0.05; ES <0.3). No significant differences were found for other analyzed variables (*p* > 0.05).

**Figure 4:**
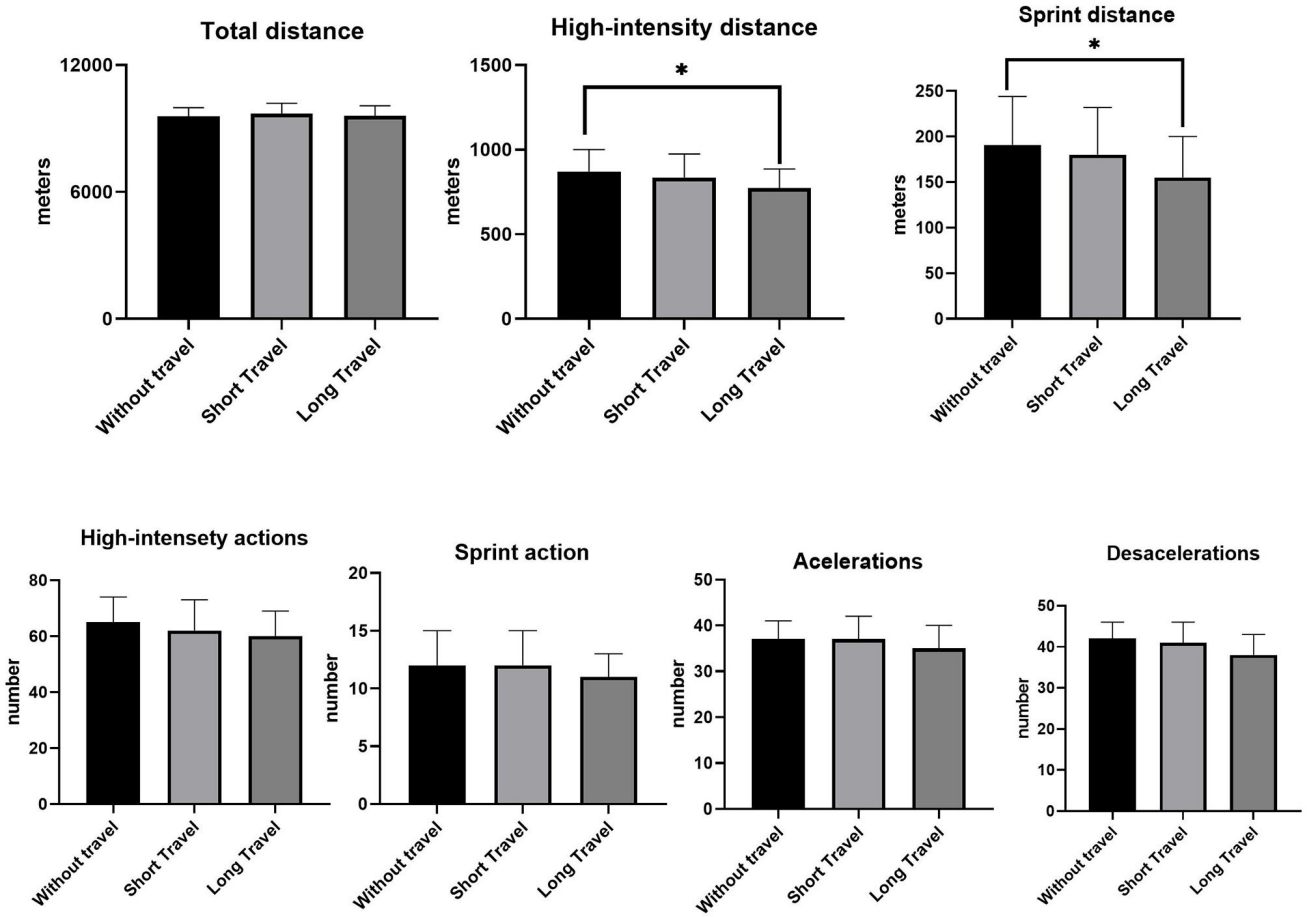
Running performance differences according to distance traveled in Brazilian elite professional soccer players. *Without travel > Long travel.

The comparisons of running performance in relation to the distance traveled to play matches are presented in Figure 4. The distance covered in sprinting and high intensity was lower in matches with long travel compared with matches without travel (*p* = 0.05; ES = 0.18; *p* = 0.04; ES = 0.2; respectively). No significant differences were found for matches with short travel (*p* > 0.05).

## Discussion

The present brief report investigated the effects of several contextual variables on running performance in professional soccer players from one club in Brazil. The main results indicated that: (i) higher TD covered was found in away matches when compared with home matches; (ii) higher TD covered was found in matches that resulted in losses when compared to draws and wins, yet higher sprinting distance was observed in matches that resulted in a draw when compared to losses; (iii) there were no effects of quality of opposition on running performance; (iv) decreased distance in high intensity and sprinting, and in the number of high intensity running and decelerations after coach replacement; and (v) higher distance covered in sprinting and high intensity in matches without travel when compared to matches with long travel.

Previous studies have suggested that the match location may cause differences in the team's strategy, which may also reflect in the physical demands during matches (Almeida et al., 2014; Aquino et al., 2017a, 2020). The results of the present brief report showed higher TD covered in away matches when compared with home matches, but the effect size was small. Contrary, previous studies performed in the 3rd and 4th divisions in Brazil showed higher values in TD covered and high-intensity activities for home matches (Aquino et al., 2017a,b, 2020).

Though, the demands imposed by the opponents might need to be considered in elite soccer. In the current study, this contextual variable did not influence the running performance of elite players. These findings contrast with previous results that showed differences in running demands when considering the quality of the opponent (Rampinini et al., 2007; Aquino et al., 2017a, 2020; Folgado et al., 2018). Though, it is worth mentioning that among the studies that observed the effects of team level, none was carried out with elite players in Brazil. Therefore, these results can be explained by the fact that matches between the teams of the elite Brazilian championship denote a largely even contest. Concerning the physical component, compared with lower leagues, no variation in physical performance has been shown, even against low-ranked opponents (Aquino et al., 2017a). Furthermore, these results also suggest that analyzing only physical variables may not fully represent players' performance during matches, and other technical, psychological and tactical factors might be relevant for match running performance. Interestingly, Folgado et al. (2014) noted that against higher-level opponents, the distance covered >19.8 km/h was greater, aiming to perform more synchronized behaviors.

In the current brief report, the players performed higher TD covered in losses than in draws and winning matches, whereas higher sprinting distance was observed in draws compared to losses. Players demonstrated similar values for high-intensity running, accelerations, and decelerations, regardless of the match outcome. These results diverged from that reported by Aquino et al. (2020) showing higher TD for draws and wins when compared with losses, and no effect on sprinting, despite analyzing matches of lower competitive standards in Brazil. Previously, Buchheit et al. (2018) observed that at the moment of the game that there was a draw, players cover greater distances above 14 km/h. The results found in the current study can be explained by the fact that during a loss situation the necessity to score goals can cause a greater physical demand when compared with winning periods when the team is in a more comfortable scoring situation (Lago et al., 2010). Indeed, in losses, the opponent team can be considered of great difficulty, which may cause greater physical demands to the players. Furthermore, in draws, the score balance during match play can cause more need to perform sprinting to create imbalance, which can increase the difficulty for the opponent's defense and create more opportunities to score (Faude et al., 2012). However, it is important to acknowledge that multidimensional aspects influence the temporary and final score of the match.

Generally, in soccer, changes of coaches during the season are very common, and Brazilian soccer is not an exception. To our knowledge, this was the first study to assess the effect of coach replacement on running performance. It is worth highlighting that coach #1 had a performance score of ~53% of wins (7 wins, 6 draws, and 4 losses), while coach #2 had a performance score of ~43% of wins (6 wins, 4 draws, and 7 losses). Previous studies showed that the change of coach would not elicit greater physical demands (Audas et al., 2002; Heuer et al., 2011). We observed differences in the distance covered in high-intensity running and sprinting, as well as in high-intensity actions and decelerations performed after coach replacement, but the effect size was small. The differences observed in running performance after coach replacement might depend on several factors, including the style of play intended by each coach. Interestingly, a previous study reported that changing the coach increased the number of points earned by the team only in the first games, meaning that the effect of coach replacement may not persist when comparing 10, 15, or 20 matches (Lago-Peñas, 2011). Hence, the immediate improvement in match performance might be explained by a higher motivational level of players in the first matches, but after a few games, this positive effect might be normalized.

Regarding the distance traveled on trips to play matches, it is worth noting that Brazil is a country of continental extension and teams have to regularly travel throughout the season. The results of the present study showed that games without travel had higher values for distances covered in high intensity and sprinting when compared to matches with long trips. No difference was found when comparing with matches with short travel. This fact suggests that long trips are likely to interfere with recovery between matches, potentially leading to declines in the ability to perform intense actions (Fowler et al., 2015a). However, it should be acknowledged that other factors related to travel, such as weather and environmental conditions, may also influence performance and recovery in soccer (Fowler et al., 2017; Lastella et al., 2019).

This brief report has some limitations, and the interpretation of the exposed results should be done with caution. The analyses were carried out considering only the matches of one season and a single team. In addition, the data referring to technical-tactical performance were not considered for analyzes. Though, in the present brief report, we observed that match location and match outcome affected the physical demands of matches in elite soccer players. Also, running performance outcomes changed with coach replacement during the season. Likewise, long traveling to play games influenced running performance during match play. These conclusions can assist coaches in planning the weekly training loads for soccer players, according to the specific requirements a match may impose.

## Data Availability Statement

The datasets generated for this study are available on request to the corresponding author.

## Ethics Statement

The studies involving human participants were reviewed and approved by State University of Rio de Janeiro. The Ethics Committee waived the requirement of written informed consent for participation.

## Author Contributions

DA: conceptualization, data curation, project administration, formal analysis, investigation, methodology, visualization, and writing—review and editing. JB: data curation, formal analysis, methodology, visualization, and writing—review and editing. RA: conceptualization, data curation, methodology, and writing—review and editing. PF: formal analysis, methodology, and data curation. FE: data curation and investigation. MT and BV: data curation and methodology. FV: conceptualization, methodology, project administration, supervision, and visualization. All authors contributed to the article and approved the submitted version.

## Funding

This research was partially supported by grants from the Carlos Chagas Filho Foundation for the Research Support in Rio de Janeiro State and Brazilian Council for the Technological and Scientific Development and was financed in part by the Coordenação de Aperfeiçoamento Pessoal de Nivel Superior—Brasil (CAPES)—Finance Code 001.

## Conflict of Interest

The authors declare that the research was conducted in the absence of any commercial or financial relationships that could be construed as a potential conflict of interest.

## Publisher's Note

All claims expressed in this article are solely those of the authors and do not necessarily represent those of their affiliated organizations, or those of the publisher, the editors and the reviewers. Any product that may be evaluated in this article, or claim that may be made by its manufacturer, is not guaranteed or endorsed by the publisher.
